# Precision Medicine for Continuing Phenotype Expansion of Human Genetic Diseases

**DOI:** 10.1155/2015/745043

**Published:** 2015-06-07

**Authors:** Hui Yu, Victor Wei Zhang

**Affiliations:** Department of Molecular and Human Genetics, Baylor College of Medicine, One Baylor Plaza, NAB 2015, Houston, TX 77030, USA

## Abstract

Determining the exact genetic causes for a patient and providing definite molecular diagnoses are core elements of precision medicine. Individualized patient care is often limited by our current knowledge of disease etiologies and commonly used phenotypic-based diagnostic approach. The broad and incompletely understood phenotypic spectrum of a disease and various underlying genetic heterogeneity also present extra challenges to our clinical practice. With the rapid adaptation of new sequence technology in clinical setting for diagnostic purpose, phenotypic expansions of disease spectrum are becoming increasingly common. Understanding the underlying molecular mechanisms will help us to integrate genomic information into the workup of individualized patient care and make better clinical decisions.

## 1. Introduction

Medical textbooks usually provide characteristic descriptions of a disease and its progression. In reality, it can be challenging to recognize clinical presentations in an individual patient due to disease variations. Besides a few cases with classical presentations, many patients have atypical or overlapping manifestations. Understanding the similarities and differences between phenotypic abnormalities of human disease remains a major task for clinicians throughout their long medical training and practice. Meanwhile, precise and individualized diagnosis is often limited by current knowledge of disease etiologies. The large number of diseases, broad and incompletely understood phenotypic spectrums, and various genetic heterogeneity are all the contributing factors that hamper the diagnostic yield. A report from a clinical laboratory specializing in diagnostic exome sequencing discovered that about 5% of individuals in their patient cohort had two definitive genetic disorders [1]. Such occurrences further complicate the traditional phenotypic-based medical evaluation approach. Failure to obtain an accurate diagnosis will likely miss a critical time window for clinical management and early intervention. Given the current wide application of molecular testing in clinical setting, identification of more than one disease in one individual is anticipated to be frequent. The establishment of a precise clinical diagnosis can also highlight the needs to examine patients for unrecognized clinical signs or presentations.

## 2. Expansion of Phenotypic Spectrum in Single-Gene Disorders

### 2.1. Clinical Heterogeneity of Single-Gene Disorders and Pleiotropic Phenotypes

Cystic fibrosis is a well-recognized single-gene disorder, and its disease severity can be roughly correlated to mutations observed in the* CFTR* gene. Unlike this well-studied disorder, patients with defects in other single genes are often found to have broad clinical presentations without well-defined genotype-phenotype correlations. For example, DNA polymerase gamma (*POLG*) deficiency can lead to multiple categories of diseases, such as progressive external ophthalmoplegia, Alpers' syndrome, Parkinsonism, and juvenile spinocerebellar ataxia-epilepsy syndrome [2]. The inheritance pattern of* POLG* related diseases is generally recessive, while autosomal dominant* POLG* mutations also exist. The clinical heterogeneity often makes diagnosis considerably difficult and contributes to diagnosis dilemmas. The clinical presentations of mitochondrial DNA (mtDNA) related disorders also show a wide spectrum of clinical variations. Despite relatively high prevalence of mitochondrial disorders, it is widely acknowledged that they are complex diseases to make a definite clinical diagnosis if based on clinical presentations exclusively [3]. Presence of inter- and intrafamily phenotypic variations further complicates clinical workout and ascertainment. While combination of family history, biochemical analyses, and pathological studies can lead to correct clinical diagnosis for some patients, a significant proportion of cases are left without molecular diagnosis. In one study, two affected siblings of a family were diagnosed initially with autosomal recessive ataxia based on clinical profile. After performing exome sequencing, a nonsense mutation in the* SURF1* gene was found segregating with the disease; therefore, the diagnosis was revised to be Leigh syndrome [4]. In another example in the same study, a family was molecularly diagnosed with* VLDLR*-associated congenital cerebellar ataxia with intellectual disability syndrome. However, the original clinical diagnosis is pontocerebellar hypoplasia since the clinical features were different from symptoms described for* VLDLR*-associated disease.

Recently, a novel clinical syndrome was described for male patients with digital abnormalities, intellectual disability, and short stature in a multigeneration family [5]. Extensive investigations including linkage studies and chromosome analyses mapped the genetic defect onto the Xp11.4-p11.21 region but were unable to associate the clinical signs and symptoms with a candidate gene. Using X chromosome targeted exome sequencing, a novel hypomorphic allele was discovered in the* EBP* gene encoding emopamil-binding protein [6]. As defects in this gene are known to cause male lethality, surviving female patients were identified most of time. Male patients carrying this hypomorphic mutation lacked typical skin and eye abnormalities found in female patients, concealing the connection of this X-linked disorder with the* EBP* gene.

### 2.2. Defining Phenotype by Specific Mutation Types

The type of genetic abnormalities can contribute to phenotype variations of a disease. A relatively large size of genomic aberration in terms of copy number variations (CNVs) is commonly seen in autosomal dominant or X-linked diseases. Point mutations are often observed in single-gene related autosomal recessive disorders. When types of point mutations range from nonsense, missense to splice site mutation, the corresponding clinical presentation can also differ considerably. Patients with nonsense mutation in alpha-1 type I collagen can have milder skeletal manifestation than these with missense mutation [7]. Missense mutations in the* MAB21L2* gene were recently identified as a new cause of eye malformations. Interestingly, both dominant and recessive inheritance patterns were observed. The patients with the monoallelic mutations showed much more severe eye abnormalities as well as defects in other systems such as skeletal dysplasia, macrocephaly, and learning disability, while others with a biallelic mutation had only a mild eye phenotype and subtle dysmorphic facial features. This evidence indicated that the zygosity of a genetic defect can contribute to different pathogenic mechanisms [8].

Currently, clinicians need to know a variety of methodologies in order to request testing for a complete molecular profile of a gene. Patients can only afford a limited number of tests due to financial burden and thus do not have the necessary genetic workup. It is now possible to obtain a relatively comprehensive genetic workup within one assay to detect both point mutation and copy number variation for a set of different genes [9, 10]. As the detection ability is expanded, we also witness growth of mutation type heterogeneities, either novel point mutations or copy number changes, leading to the phenotype expansion of human disorders [6, 11].

### 2.3. Phenotype Modified by Allelic Interactions

The penetrance of a disease phenotype can be influenced by combinatory effect of two alleles. Most recently, sequencing a cohort of patients with sporadic congenital scoliosis identified multiple heterozygous null mutations in* TBX6* gene [12]. The discordant interfamily phenotypes of carriers bearing deleterious mutations led to the interrogation of the role of* TBX6* haplotype in this disease. After extensive linkage and genotyping analysis, it was revealed that copresence of a* TBX6* null allele and a common haplotype containing three common single-nucleotide polymorphisms (SNPs) can lead to congenital scoliosis in patient. Thus, this common* TBX6* haplotype acts as a hypomorphic genetic modifier to modulate the penetrance of the disease presentation in carriers of* TBX6* null mutations.

## 3. Increased Genetic Heterogeneity for Defined Phenotypes

Genetic heterogeneity is one of the important factors to consider during genetic workup for disorders with overlapping clinical presentations. For disorders with relatively defined clinical phenotypes, such as glycogen storage disorder, both clinical evaluation and routine laboratory testing may provide initial clues leading to a correct clinical diagnosis. Regarding disorders with nearly indistinguishable clinical phenotypes, such as congenital defects of glycosylation, the result from biochemical testing may not be sufficient to narrow down a possible underlying genetic cause. As to disorders with a broad spectrum of clinical presentations, the large number of genes associated with these phenotypes makes it difficult or impossible to pinpoint an exact gene if molecular testing is not requested. Disease-targeted multigene panels and whole-exome sequencing allow precise molecular diagnosis made for patients with previously unrealized genetic defects [13, 14]. More importantly, a genomic approach will help to understand the molecular mechanism and also provide valuable information for diagnosis of exclusion.

## 4. Epistatic Interactions Modulating Clinical Presentations

Epistatic interactions have been recognized to play important roles in pathogenesis and phenotype variability [15]. When more genes are included for molecular diagnosis, identification of more than one genetic disease or multiple carrier mutations status will be higher for our patients. Some of those mutated genes belong to a common molecular pathway. The documented epistatic interaction is ciliopathy caused by genes related to Bardet-Biedl syndrome [16]. Further studies have revealed that synergistic mutations can involve more than two BBSs genes in patients with this syndrome. Mutant load in these BBSs loci appears to be associated with the severity of clinical phenotype [17]. So far, about 18 genetic loci have been identified as responsible for this disorder [18]. While a phenotype can be influenced by the presence of other modifier genes, it is hard to evaluate their effects without obtaining genotype information for all the interrelated genes [19]. With wide application of comprehensive genetic testing, we will have better opportunities to define the contribution of epistatic mutations to clinical presentation in these patients. Understanding of these epistatic interactions will help us to elucidate the disease complexity and uncover additional phenotypes [20].

## 5. Shift of Phenotype- to Genotype-Based Clinical Practice

The rapid progress of high-throughput genomic sequencing and corresponding analysis tools in molecular diagnosis have revolutionized the practice of clinical diagnosis [21]. Comprehensive genomic evaluation can deliver accurate results in a more cost-effective manner. One such example is the adaption of chromosomal microarray (CMA) in a clinical setting. This method has been gradually replacing G-banded karyotyping as a first-tier clinical diagnostic test for patients with suspected developmental disabilities or congenital anomalies [22]. High-throughput sequence-based molecular diagnosis assay is another emerging first-tier test [23]. Diagnosis of genetically or clinically heterogeneous disorders, which have been historically proven to be challenging, is turning out to be relatively straightforward and more accurate nowadays. As the detection ability and yield of molecular diagnosis improve and the knowledge of disease presentation and corresponding molecular mechanism is also expanding, clinicians will be able to rely on objective and unbiased genetic evidences to make a definite diagnosis.

## 6. Summary

Ending the diagnostic odyssey, the FORGE (Finding of Rare Disease Genes) Canada Consortium identified genetic causes for 146 disorders over a 2-year period through a nation-wide collaboration effort among clinicians and scientists [24]. Precision medicine has a significant impact on medical knowledge and will lead us to genetics evidence based medicine and improve the overall population health. More clinical research and pharmaceutical development will be warranted by the increased understanding of the underlying causes of such diseases [25]. However, growth of both phenotypic and genetic knowledge of human diseases imposes a great informatics challenge. Structured phenotype matching procedure needs to be implemented to facilitate the diagnostic decision. The Human Phenotype Ontology (HPO) project was developed to integrate phenotype information across scientific fields and databases and provide standardized phenotype annotations [26]. Another aspect of precision medicine is individually tailored therapy and care management. Personalized pharmacogenetic information of drug response phenotype can be incorporated into the treatment selection [27–30]. More ideally, new drugs can be developed based on a person's genetic profile by bridging molecular diagnosis and pharmaceutical research, and potential therapeutic targets can be evaluated systematically in the context of an interconnected biological network [31].
